# Chemical Profile and Bioactivities of Three Species of *Mentha* Growing in the Campania Region, Southern Italy

**DOI:** 10.3390/plants14030360

**Published:** 2025-01-24

**Authors:** Rosaria Francolino, Mara Martino, Filomena Nazzaro, Carmina Sirignano, Florinda Fratianni, Francesca Coppola, Laura De Martino, Carmen Formisano, Vincenzo De Feo

**Affiliations:** 1 Department of Pharmacy, University of Salerno, Via Giovanni Paolo II, 132, 84084 Fisciano, SA, Italy; rfrancolino@unisa.it (R.F.); martinomara966@gmail.com (M.M.); defeo@unisa.it (V.D.F.); 2 Institute of Food Sciences, CNR, 83100 Avellino, Italy; mena@isa.cnr.it (F.N.); florinda.fratianni@isa.cnr.it (F.F.); fracop93@gmail.com (F.C.); 3 Department of Pharmacy, School of Medicine and Surgery, University of Napoli Federico II, 80131 Napoli, Italy; carmina.sirignano@unina.it

**Keywords:** *Mentha*, hydroalcoholic extract, chemical composition, antioxidant, antibacterial

## Abstract

The genus *Mentha* (Lamiaceae), comprising aromatic perennial plants widely distributed in temperate regions, holds significant medicinal and commercial value. This study aimed to investigate the chemical profile and bioactivities of hydroalcoholic extracts from *Mentha longifolia* (L.) L., *Mentha pulegium* L., and *Mentha spicata* L. harvested from the Campania region, Southern Italy. Chemical analysis using LC-HRESIMS/MS identified a total of 21 compounds. The extracts, particularly *M. pulegium*, exhibited notable antioxidant activity, evaluated through DPPH and FRAP assays, probably related to their chemical composition. Both *M. pulegium* and *M. longifolia* demonstrated a higher phenolic content, with *M. pulegium* also containing the highest levels of flavonoids. In addition, the extract’s ability to inhibit biofilm formation was evaluated against several pathogenic strains, including Gram-positive bacteria (*Listeria monocytogenes* and *Staphylococcus aureus*) and Gram-negative bacteria (*Acinetobacter baumannii*, *Pseudomonas aeruginosa*, and *Escherichia coli*) using crystal violet and MTT assays. All extracts effectively inhibited biofilm formation in *A. baumannii* and *P. aeruginosa*, with *M. pulegium* also showing moderate activity against the metabolism of *L. monocytogenes*. The pronounced antibacterial and biofilm-inhibitory properties of *M. pulegium* highlight its potential for pharmaceutical applications.

## 1. Introduction

The growing interest in secondary metabolites stems from their notable anti-microbial and antioxidant properties, highlighting their potential use in both the food and pharmaceutical sectors. These natural compounds could be an alternative to synthetic antioxidants, which have been reported to present risks to human health due to their side effects [1]. In this context, natural products can improve food stability and preservation by inhibiting the growth of foodborne bacteria and pathogens, while also offering protection against oxidative damage [2]. Several studies in the literature have demonstrated that natural antioxidants show a diverse array of bioactivities, demonstrating antibacterial, antiviral, antithrombotic, anti-inflammatory, anti-allergic, and vasodilatory effects [3]. The main secondary metabolites responsible for these pharmacological and antioxidant activities are phenolic compounds, including flavonoids [4]. These compounds are generally considered less toxic compared to synthetic alternatives like BHA (butylated hydroxyanisole) and BHT (butylated hydroxytoluene) [5]. The antioxidant properties of these metabolites stem from their redox characteristics, enabling them to function as reducing agents, hydrogen donors, and metal ion chelators [5].

*Mentha* (Lamiaceae family), a genus of aromatic perennial plants distributed in temperate regions of Europe, Asia, Australia, and South Africa, holds significant medicinal and commercial importance [6]. The genus includes 42 species, 15 hybrids, and hundreds of subspecies and cultivars [7]. Plants of this genus have been extensively studied for their diverse activities, including anti-inflammatory, sedative, antioxidant, antibacterial, and antifungal effects, along with many folk usages [8]. These biological activities are primarily attributed to phenolic diterpenes, flavonoids, and phenolic acids [9] that are present in the leaves, flowers, seeds, and bark of these plants [10].

*Mentha longifolia* (L.) L., *Mentha pulegium* L., and *Mentha spicata* L. are among the most widespread and significant species of the genus in the Mediterranean region, where they are well adapted to the favorable climatic conditions and are commonly employed in traditional medicine and for culinary uses [8,11].

*M. longifolia*, commonly known as horse mint, like other members of the *Mentha* genus, is used in folk medicine and gastronomy. The leaves are employed in the treatment of fever, gastric disorders, and headaches. Additionally, this species exhibits diuretic, stomachic, digestive, carminative, antibacterial, and anti-inflammatory activities [12].

*M. pulegium*, also known as pennyroyal, is used in gastronomy, perfumery, and in the pharmaceutical industry. The aerial parts of the plant are employed in traditional medicine for their carminative and antispasmodic effects, particularly in treating intestinal issues [13].

The dried or fresh leaves of *M. spicata* (spearmint) have several medicinal and culinary uses. Spearmint is commonly used as a flavor enhancer and herbal tea. Furthermore, it is used in traditional medicine in the treatment of fever, biliary disorders, stomach pain, menstrual cramps, and gingivitis due to its carminative, stimulating, antispasmodic, and diuretic properties [14].

Due to the widespread use of plants from the *Mentha* genus, there are numerous studies in the literature regarding their essential oils [15,16]. However, the number of studies focusing specifically on the hydroalcoholic extracts of this genus is relatively limited compared to the extensive body of literature available on their essential oils. This work aimed to compare three hydroalcoholic extracts from *M. longifolia*, *M. pulegium*, and *M. spicata* collected in the Campania region, Southern Italy. The extracts were analyzed to compare (i) their chemical compositions using LC-MS and the total contents of phenols (TPC) and flavonoids (TFC); (ii) their antioxidant properties employing DPPH (1,1-diphenyl-2-picrylidrazyl) and FRAP (ferric-reducing antioxidant power) tests; (iii) their potential antibacterial activity using MTT and crystal violet assays against selective Gram-positive (*Listeria monocytogenes* and *Staphylococcus aureus*) and Gram-negative (*Acinetobacter baumannii*, *Pseudomonas aeruginosa*, and *Escherichia coli*) pathogens.

## 2. Results and Discussion

### 2.1. Chemical Analysis

The analysis of the extracts using LC-HRESIMS/MS led to the identification and classification of 21 components, which are categorized into various molecular classes (Table 1). The missing peak areas in Table 1 are due to the specific parameters set in the analytical software. While the compounds are visible in the chromatographic runs of hydroalcoholic extracts, they could not be quantified because the software parameters, such as intensity thresholds or integration settings, were optimized for the most abundant peaks. These settings may have excluded lower-intensity signals or those outside the predefined criteria. Adjusting the parameters might allow for the quantification of these compounds; however, this was not the focus of the current analysis, which prioritized the most prominent components for comparative purposes.

Compound **1** was recognized as chlorogenic acid (C_16_H_18_O_9_) according to the [M + H]^+^ ion at *m*/*z* 355.1021 and its fragmentation pattern analysis, which showed a fragment at *m*/*z* 163.0387, related to the loss of quinic acid, which is consistent with previously published data [17]. This compound has been identified in several *Mentha* species [18,19].

Compound **2** gave an [M + H]^+^ ion at *m*/*z* 227.1275, which was attributed to tuberonic acid; the MS^2^ fragments at *m*/*z* 209.1171 and *m*/*z* 191.1066 are related to the subsequent neutral loss of H_2_O [20].

Compounds **3**, **4**, **5**, **6**, and **7** belong to the class of flavone-*O*-glycosides. Compounds **3** and **4** gave [M + H]^+^ ions at *m*/*z* 595.1645 and *m*/*z* 579.1694, which were attributed to luteolin-7-*O*-rutinoside and apigenin-7-*O*-rutinoside, respectively. The analysis of the MS^2^ spectra showed a fragment at *m*/*z* 449.1080 for **3** and a fragment at *m*/*z* 433.1116 for **4**, both due to the loss of a rhamnose moiety. Other fragments were detected at *m*/*z* 287.0552 and *m*/*z* 271.0594, corresponding to the protonated form of luteolin for compound **3** and apigenin for compound **4**. This fragmentation pattern has been documented in previous studies in *M. pulegium* by Taamalli and coworkers [19] and El Gazar and coworkers [20].

Diosmin (diosmetin 7-*O*-rutinoside) (compound **5**), which has been found in several species of *Mentha* [19], gave a protonated ion [M + H]^+^ at *m*/*z* 609.1782, corresponding to the molecular formula C_28_H_32_O_15_. The MS^2^ spectra displayed daughter ions at *m*/*z* 463.1234 and *m*/*z* 301.0706, which were generated by the elimination of a deoxyhexose unit (146 amu) and subsequently the leak of a hexose moiety (162 amu). This finding aligns with previously published data [20].

The other two flavone-*O*-glycosides, linarin (acacetin 7-*O*-rutinoside) (**6**) and di-dymin (**7**), gave an [M + H]^+^ ion at *m*/*z* 593.1851 and *m*/*z* 595.2007, respectively. Linarin MS^2^ spectra revealed *m*/*z* 447.1282 and *m*/*z* 285.0754 as fragment ions, which were generated by the leak of the deoxyhexose unit (146 amu), followed by the loss of a hexose moiety (162 amu), thus resulting in the detection of the aglycone (*m*/*z* 285.0754), as previously reported [20]. The analysis of didymin, based on the MS^2^ data, revealed a fragment at *m*/*z* 287.0917, related to the consecutive leak of rhamnose and glucose molecules, which was also confirmed in the literature [21].

Compound **8** gave an [M + H]^+^ ion at *m*/*z* 341.0652, attributed to salvianolic acid G; the MS^2^ fragments at *m*/*z* 323.0551 and *m*/*z* 297.0764 were related to the loss of H_2_O and CO_2_, respectively. This fragmentation was also reported previously in *M. haplocalyx* Briq [22].

Compound **9** was attributed to kaempferol on the basis of the [M + H]^+^ ion at *m*/*z* 287.0548 and the MS^2^ spectra. The detected fragments at *m*/*z* 269.0428, *m*/*z* 245.0447, and *m*/*z* 153.0436 were related to the loss of H_2_O, CH_2_O, and C_8_H_6_O_2_, respectively, in agreement with the pathway of fragmentation of this flavonoid, as was previously reported in some species of the genus [23].

Compound **10** produced an [M + H]^+^ ion at *m*/*z* 331.0810, assigned to the flavonoid ja-ceosidin, which was previously reported in *M. pulegium* [24]. The compound gave the fragment ion at *m*/*z* 316.0580, which was consistent with the leak of the CH_3_ group (15 amu), followed by fragment ions at *m*/*z* 303.0868 and *m*/*z* 288.0623, derived from the loss of the CO group (28 amu) and from C_2_H_3_O. This pathway of fragmentation was also reported in *Mentha haplocalyx* Briq. [22].

The flavonoid naringenin, compound **11**, was assigned to the ion at *m*/*z* 273.0757 [M + H]^+^. The compound produced the fragment ion at *m*/*z* 165.0544, due to the loss of the C_6_H_4_O_2_ group, followed by fragment ions at *m*/*z* 130.0039, derived from the loss of the C_7_H_11_O_3_ group.

Compounds **12** and **15** are two flavonoids that yield [M + H]^+^ ions at *m*/*z* 361.091. The distinction between these compounds is attributed to the varying relative abundances of the daughter ions produced. Specifically, one compound corresponds to sideritiflavone and the other to thymonin, based on MS^2^ fragmentation analysis. Sideritiflavone generates a fragment ion at *m*/*z* 346.0676, which is consistent with the leak of a CH_3_ group, followed by fragment ions at *m*/*z* 331.0445 and *m*/*z* 328.0576, resulting from the loss of CH_3_ and CH_5_O groups, respectively. Notably, the MS^2^ data analysis revealed identical abundances of daughter ions, consistent with findings by Xu and colleagues in 2017 [22]. In contrast, the analysis of thymonin via MS^2^ data unveiled a fragment at *m*/*z* 346.0682 linked to the removal of a methyl group, followed by two fragment ions at *m*/*z* 331.0445 and *m*/*z* 313.0340, with consistent abundance, as documented in the literature by Xu et al. [22]. Zaidi and coworkers [24] reported the presence of both compounds in all three *Mentha* species.

Compound **13** was assigned to the flavonoid diosmetin, which had already been found in *M. pulegium* [20], giving the [M + H]^+^ ion at *m*/*z* 301.0704. The fragmentation pattern was corroborated by the existing literature [25].

Compound **14** generated an [M + H]^+^ ion at *m*/*z* 345.0960, attributed to flavonoid xhantomicrol, as previously reported in the all three *Mentha* species [24]; the MS^2^ fragments at *m*/*z* 330.0727 and 301.0701 were related to the loss of CH_3_ and C_2_H_2_O, respectively, in agreement with the fragments reported in other *Mentha* species [22].

Compound **16** displayed an [M + H]^+^ ion at *m*/*z* 375.1068, attributed to hymenoxin, as previously reported in the all three species [24]; the MS^2^ analysis showed three ions at *m*/*z* 360.0841, 345.0608, and 342.0735, in agreement with the pathway of fragmentation of this flavonoid, as has already been reported in this genus [26].

The flavonoid nobiletin (**17**) was previously reported in *M. longifolia* [27]. This assignment was performed on the basis of the [M + H]^+^ ion at *m*/*z* 403.1376, as well as the fragment ions at *m*/*z* 388.1154 and 373.0897, which agreed with the leak of the CH_3_ groups [21].

Compound **18** generated an [M + H]^+^ ion at *m*/*z* 287.0916, which was attributed to flavonoid sakuranetin, as has previously been reported in the *Mentha* genus [28]. The fragment ion at *m*/*z* 167.0342 was in agreement with the formation of a C_8_H_7_O_4_ group, as reported by Pavlešić et al. [29].

The flavonoid nevadensin (**19**) displayed an [M + H]^+^ ion at *m*/*z* 345.0962. This compound has previously been reported in *M. longifolia* [22]. The MS^2^ spectrum revealed the diagnostic ions at *m*/*z* 312.0623, 330.0738, and 315.0504. Moreover, the ion at *m*/*z* 135.0440 was also registered, which was in agreement with the pathway fragmentation of ring ^0,2^B^+^ [30].

Compound **20** produced an [M + H]^+^ ion at *m*/*z* 359.1122, attributed to 5-dydroxy-6,7,3′,4′-tetramethoxy flavone. The MS^2^ analysis showed some ions at *m*/*z* 326.0788, 344.0893, 315.0871, and 298.0840, which were previously reported in an aqueous extract of *M. haplocalyx* Briq [22,26].

The [M + H]^+^ ion at *m*/*z* 389.1221 was assigned to flavonoid 5-hydroxyauranetin (**21**). The MS^2^ analysis indicated the presence of some diagnostic ions at *m*/*z* 374.0990, 359.0758, 356.0883, 341.0650, and 328.0933, which were related to the loss of methyl groups and water, as previously reported in an aqueous extract of *M. haplocalyx* Briq [22,26].

According to the LC–MS/MS investigation of this study, it was shown that the profiles and contents of phenolic compounds vary depending on the crude extract of the plant.

*M. longifolia* exhibited a more complex profile, containing 20 compounds, which includes 17 flavonoids and 3 phenolic acids. Among these, moderate amounts of diosmetin were detected, while low amounts of nobiletin were observed. Diosmetin is classified as an O-methylated flavone and is also found in this extract as the aglycone part of the flavonoid glycoside diosmin.

Thymonin and nevadensin, both of which are trimethoxyflavones, appeared as the predominant compounds. In addition, the trihydroxyflavone jaceosidin and the glycoside linarin are unique to the extract of *M. longifolia*.

The analysis of the extracts of *M. pulegium* and *M. spicata* showed a total of 17 compounds. The extract of *M. pulegium* contains 14 flavonoids, with thymonin being the least abundant compound; nevadensin is identified as the most abundant constituent in this extract. 5-Hydroxy-6,7,3′,4′-tetramethoxy flavone was present exclusively in this extract. *M. spicata* extract contained 15 flavonoids, among which sideritiflavone was the most abundant flavonoid. In this case, chlorogenic acid was the least present compound and salvianolic acid was completely absent. Therefore, the phenolic fraction of this extract is less prominent compared to that of the other two extracts.

### 2.2. Determination of Total Phenolic Content, Total Flavonoid Content, and Antioxidant Activity

**Figure 1:**
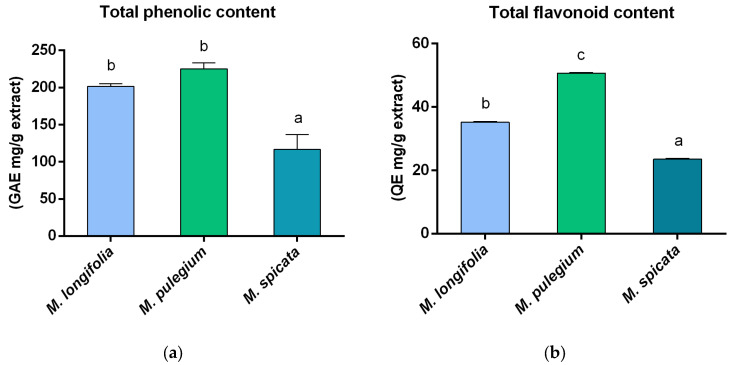
(**a**) Total phenolic and (**b**) flavonoid contents in *M. longifolia*, *M. pulegium*, and *M. spicata*. GAE = gallic acid equivalents. QE = quercetin equivalents. Values were expressed as mean ± SD from three independent experiments. Different letters within the same graph indicate significant differences at *p* < 0.05 (two-way ANOVA followed by Tukey’s post hoc test).

The total phenolic content (TPC) was estimated using the Folin–Ciocalteu spectrophotometric test. The result was reported as mg GAE (gallic acid equivalents)/g extract. As represented in Figure 1a, the highest TPC was found in *M. pulegium* (224.88 mg GAE/g). The total flavonoid content (TFC) was assessed using the aluminum chloride colorimetric test. The value was expressed as mg QE (quercetin equivalent)/g extract. The highest TFC was 50.67 mg QE/g in the *M. pulegium* extract (Figure 1b). The TFC constituted 17% of *M. longifolia*, 22% of *M. pulegium*, and 20% of *M. spicata* in relation to the total phenolic content.

The antioxidant power of the hydroalcoholic extracts was carried out employing two spectrophotometric analyses: DPPH and FRAP. The two methods evaluate a substance’s ability to donate hydrogen (DPPH) and its action as a reducing agent (FRAP). The DPPH radical exhibits an absorption peak at 517 nm, which diminishes when exposed to antiradical substances that can donate hydrogen. In the FRAP test, the reduction of the complex from Fe(III)-2,4,6-tripyridyl-s-triazine (also known as [Fe(III)-(TPTZ)^2^]^3+^) to Fe(II), [Fe (II)-(TPTZ)^2^]^2+^ causes a color change to navy blue. The reaction can be monitored spectrophotometrically at a wavelength of 593 nm. The data in Table 2 show the quantity of each hydroalcoholic extract needed to decrease DPPH absorbance by 50% and the quantity of equivalent Fe^2+^ reduced/g of extract.

Among secondary metabolites, phenolic compounds are a crucial class known for their antioxidant activity. The Folin–Ciocalteu (FC) assay is commonly used to estimate the total phenolic content (TPC) in plant extracts by measuring their reducing capacity, as phenolic compounds reduce the FC reagent, causing a color change from yellow to blue. However, the assay is not specific to phenolics and can also react with other reducing agents like sugars and proteins, potentially leading to an overestimation of phenolic content. Despite these limitations, the FC assay is valuable for comparing the relative antioxidant capacities and extraction yields of different samples, providing insights into their overall reducing abilities [31].

These phenols carry out their antioxidant effect through the scavenging action of free radicals, primarily due to their hydroxyl groups. Among these compounds, flavonoids are particularly noteworthy for their free radical scavenging properties, as well as their antibacterial and anti-inflammatory properties [5]. In this study, the total flavonoid content was determined using a method suitable for the diverse flavonoids present in the samples, including various glycosides and methoxy-substituted flavonoids, as it offers enhanced sensitivity and broader applicability compared to the aluminum chloride method, which is selective for certain flavonoid subclasses. Consequently, the selected method provides a more accurate representation of the total flavonoid content in the extracts, aligning with the study’s objective of quantifying a wide range of flavonoids [31]. The total phenolic content was highest in the *M. pulegium* and *M. longifolia* extracts, with *M. pulegium* also demonstrating the major flavonoid levels among the three extracts. These elevated levels of phenolics and flavonoids contributed to a stronger antioxidant activity [32], as demonstrated in both the DPPH and FRAP assays. In fact, the *M. pulegium* and *M. longifolia* extracts showed a greater antioxidant power than *M. spicata* in the DPPH assay (Table 2), and *M. pulegium* also exhibited a higher antioxidant power in the FRAP test (Table 2).

There are few studies in the literature on the hydroalcoholic extracts of these plants. Some studies performed on non-hydroalcoholic extracts of *M. longifolia*, *M. pulegium*, and *M. spicata* exhibited a lower phenol content [5,14,33]. The phenolic content in the methanolic extract of *M. longifolia* reported by Raj et al. [5] was lower than that of the hydroalcoholic extract here reported, while the total flavonoid content was comparable. Moreover, the hydroalcoholic extract analyzed by Ebrahimzadeh and collaborators [34] also exhibited a lower phenol and flavonoid content. No studies in the literature have investigated the antioxidant activity of the hydroalcoholic extract of *M. longifolia*.

In the literature, the hydroalcoholic extract of *M. spicata* showed a higher phenolic content and a lower IC_50_ value compared to the extract analyzed in this study [35]. In the work by Mata et al. [33], the ethanolic and water extracts of *M. spicata* contained lower levels of phenols than the hydroalcoholic extracts assessed here. In line with this, Fatiha and coworkers [14] observed lower phenolic and flavonoid contents in the ethanolic extract of *M. spicata*.

Mata and co-workers [33] demonstrated that the ethanolic and water extracts of *M. pulegium* had lower phenol contents than the hydroalcoholic extracts analyzed in this work, while in another work, both the phenol and flavonoid contents of the ethanolic extract of *M. pulegium* were lower than those observed in our extracts [14]. Tacherfiout and collaborators [36] observed that the hydroalcoholic extract of *M. pulegium* had lower phenol and flavonoid contents but still had a good antioxidant and reducing capacity.

The major constituent in *M. longifolia*, and the second most abundant in *M. spicata*, is diosmetin, which was investigated by Bai and colleagues [37] for its antioxidant activity. Their study revealed that diosmetin exhibits a moderate DPPH radical scavenging activity. Conversely, nevadensin, the main component in *M. pulegium*, which is also found in significant amounts in *M. longifolia*, has demonstrated limited antioxidant capability. Ganapaty and coworkers reported a poor antioxidant performance for nevadensin [38]. Santaflavone, identified only in *M. pulegium* as its second major component, was reported for its antioxidant action [39]. This compound, along with other constituents, could play a central role in the strong antioxidant properties observed for the hydroalcoholic extract. Another of the main compounds, xanthomicrol, the third most prevalent compound in *M. pulegium*, was reported for its potent DPPH scavenging capability and potential reducing power [32]. It was also present in *M. spicata* in smaller quantities, as well as in similar concentrations in *M. longifolia*. In *M. spicata*, the main component was sideritiflavone, which Mohamadi et al. reported as an effective antioxidant with activity comparable to vitamin C [40]. Finally, *M. spicata* also contained hymenoxin as a primary component, a compound reported for its significant antioxidant properties [41]. Hymenoxin was also abundant in *M. longifolia*, though it was found in smaller amounts in *M. pulegium*.

### 2.3. Antibiofilm Activity

The assessment of the antibiofilm capability was conducted through the crystal violet test and the MTT test on mature biofilms, adding 10 and 20 µL/mL of extracts, previously resuspended in DMSO, 24 h after the start of bacterial growth when the biofilm is considered mature and the sessile cells are protected by the scaffolding of extracellular material from the external environment and physical, chemical, and biological agents (including antibiotics). The choice of using 10 and 20 µL/mL of extracts was made based on the data of the MIC test, conducted with resazurin. The data are reported in Table 3 (MIC) and Table 4 (antibiofilm tests).

The crystal violet assay showed a remarkable antibiofilm effect exhibited by the studied extracts on the *A. baumannii* strain. This action consistently surpassed 42.95% at 10 μL/mL, and reached an impressive inhibition percentage of 50.78% at 20 μL/mL. The three samples, at the highest dose, exhibited biofilm inhibitory capability, albeit less effectively, against *P. aeruginosa*. These data could have important implications for the development of antibiofilm strategies. No similar inhibitory action was found against *L. monocytogenes* and *P. aeruginosa* (apart from the *M. pulegium* extract at the highest concentration used). However, the extracts were found ineffective against *E. coli* and *S. aureus*. Through the MTT test, it was possible to evaluate whether the extracts were able to act by inhibiting the metabolism of the sessile cells present within the bacterial biofilm.

The MTT test highlighted that the inhibitory action does not also translate into their inhibitory efficacy on the metabolism of its sessile cells, but that there are probably other mechanisms or processes that could lead to a blockage of the mature biofilm by up to 50%. As for the crystal violet test, the negative results obtained from the MTT test confirmed that the extracts were ineffective in counteracting the metabolic processes of the sessile cells of *E. coli* and that their inhibitory biofilm action also translates into metabolic inhibition.

The MTT test also demonstrated that a correspondence of inhibitory action was also noted in the case of *P. aeruginosa*, with the extracts of *M. pulegium* and *M. spicata*. Indeed, the inhibitory efficacy of the *M. spicata* extract monitored in the crystal violet test (21.54%) was greater in the MTT test (37.71%), proving that this extract is capable of acting above all on the metabolism of the sessile cells of *P. aeruginosa*. However, the *M. longifolia* extract, which had also determined an inhibition of 22.67%, resulted inactive on the sessile metabolism of that strain. In the case of *L. monocytogenes*, where the *M. pulegium* extract determined an inhibitory effect on its biofilm, it did not, on the contrary, prove active in inhibiting the biofilm of the sessile cells. Instead, the *M. spicata* extract, ineffective in acting on the biofilm of this pathogenic strain, manages to inhibit (up to 37.71%) the metabolism of its sessile cells. In addition to *E. coli*, the most resistant strain to the three extracts was *S. aureus*, with the exception of its sensitivity to the *M. pulegium* extract, whose presence in the microbial culture medium resulted in a 9.51% inhibition of the metabolism of its sessile cells.

The three extracts did not exhibit the same activity on biofilm. *Mentha longifolia*, for instance, was ineffective against *S. aureus* but effective against *P. aeruginosa*. Our results are only in partial disagreement with those recently obtained by Kazmi et al. [42], who found a significant antibiofilm activity against *P. aeruginosa* and *S. aureus*, as exhibited by a boiled water extract from *M. longifolia* powder. However, such discordant behavior could probably depend on the different extraction methods and the different plant origin. Our results also agree with Quave et al. [43], who did not observe the antibiofilm activity of the ethanolic extracts of *M. spicata* and *M. pulegium* against *S. aureus*. The antibiofilm activity shown by all the extracts against *A. baumannii* in the crystal violet and MTT tests is relevant. Following the World Health Organization, this pathogen strain belongs to the so-called “critical group” of pathogens, which are the pathogens with the highest warning to public health and with an increasing trend for antimicrobial resistance [44]. Our results evidenced that the extracts act on the mature biofilm, which is a more complex situation than that of the immature biofilm (slightly more easy to eradicate), which could lead to the targeting of segments of people, such as the elderly and infants. The action occurs both on the biofilm and on the metabolism of sessile cells when bacterial virulence increases dramatically. Our work evaluated, for the first time, the efficacy of the three *Mentha* extracts to fight the metabolic changes occurring in the metabolism of the sessile cells. The circumstance that all three extracts inhibited the metabolism of sessile cells can be considered a promising result. As highlighted in the scientific literature, the processes of metabolic modification within the biofilm ’niches’ are significant threats to host health. Understanding and potentially controlling these processes could lead to a significant reduction in pathogen virulence and a strengthening of the body defenses against bacterial infections [45].

## 3. Materials and Methods

### 3.1. Plant Material and Extract Preparation

*Mentha spicata*, *Mentha pulegium*, and *Mentha longifolia* were gathered from the Campania region, specifically in the Salerno province, in June 2022. Voucher specimens for each species (DF 25/2024, DF 26/2024, and DF 27/2024) were preserved in the Herbarium of the Medical Botany Chair, Department of Pharmacy, Salerno University. The plant leaves were cleaned and air-dried. Approximately 180 g of dried leaves per species was macerated with 70% ethanol (*v*/*v*). This procedure was conducted three times, using fresh solvent each time, with a maceration period of 5 days per cycle. The extracts were filtered, and the solvent was removed to yield the dry extracts. The final hydroalcoholic extracts yielded 7% for *M. longifolia*, 4% for *M. spicata*, and 12% for *M. pulegium*.

### 3.2. Chemical Analysis: LC-HRMS and LC-HRMS2

The LC-HRESI-MS/MS analysis of the hydroalcoholic extracts was performed using a Thermo LTQ Orbitrap XL mass spectrometer (Thermo Fisher Scientific Spa, Rodano, Italy) equipped with an ESI-MAX source, paired with a Thermo U3000 HPLC system (Agilent Technology, Cernusco sul Naviglio, Italy). Chromatographic separation was carried out on a Kinetex Polar C18 column (100 × 3.0 mm; 100 Å; 2.6 µm). The volume of injection was set at 5 µL with a flow rate of 0.5 mL/min, using a mobile phase comprising solvent A (0.1% formic acid in water, *v*/*v*) and solvent B (acetonitrile). A linear gradient was employed, starting with 5% solvent B and increasing to 95% over 15 min, followed by a 5 min hold at 95% B. HRMS and MSn spectra were acquired in positive ionization mode with data-dependent acquisition, fragmenting the five most intense peaks per scan. The parameters of spray voltage, capillary voltage, gas flow, and capillary were reported in a previous paper [46]. The acquisition range spanned *m*/*z* 150–1500.

### 3.3. MZmine Data Processing

Raw MS data were processed using Mzmine 3.4.27 with adjustments to the method outlined by Heuckeroth et al. [47]. Centroid mass detection was applied to both MS1 and MS2 levels. Parameters for the ADAP chromatogram builder, including the minimum number of consecutive scans, minimum intensity for consecutive scans, minimum peak intensity, and *m*/*z* (mass-to-charge ratio) tolerance, were carefully configured. The wavelet algorithm was used for the local minimum feature resolver, with optimized settings for the signal-to-noise (S/N) threshold, intensity window S/N, minimum feature height, coefficient area threshold, peak duration range, and retention time (RT) wavelet range. Chromatograms were deisotoped using a ^13^C isotope filter, adjusting the *m*/*z* and RT tolerances, with a maximum charge of 1 and a selection of the most intense isotopes as representatives. Additional steps included identifying isotopic peaks, aligning features with the join aligner, and gap filling for peak detection. The final parameters for each step in the workflow are detailed in Supplementary Information Table S1. The resulting feature list was exported as a .csv file containing RT, *m*/*z*, and peak area information for the three analyzed samples.

### 3.4. Determination of Total Phenolic Content, Total Flavonoid Content, and Antioxidant Activity

#### 3.4.1. Analysis of Total Phenol and Flavonoid Content

The total phenolic content (TPC) was determined using the Folin–Ciocalteu test [48], with some modifications [46]. A calibration curve was prepared with several concentrations of gallic acid (from 0.05 to 0.5 mg/mL). TPC was reported as mg of gallic acid equivalent per gram of extract. The experiment was performed three times, and results were expressed as the mean ± SD.

Total flavonoid content (TFC) was evaluated using the aluminum chloride colorimetric method, as described by Baba and Malik [49], with some modification [46]. The quercetin calibration curve covered concentrations between 0.0625 and 0.5 mg/mL. TFC was expressed as mg of quercetin equivalent per gram of extract. Results, derived from three replicates, were reported as mean ± SD.

#### 3.4.2. Antioxidant Activity

##### DPPH Assay

Hydroalcoholic extract aliquots were dissolved in methanol to achieve a final concentration range of 12.5 to 1000 µg/mL. The antiradical activity was assessed using the stable DPPH radical (1,1-diphenyl-2-picrylhydrazyl) following the method of Lee et al. [50], with slight modifications, as previously reported [46]. The data were reported as IC_50_ values, representing the extract concentration needed to lower the DPPH absorbance by 50%. Ascorbic acid was employed as the reference standard. The assay was carried out three times, and the findings were reported as mean ± SD.

##### FRAP

The Ferric Ion Reduction Antioxidant Power (FRAP) assay was performed following the method previously reported [46]. The assay measures the reducing power of a sample by evaluating its ability to reduce the Fe^3^⁺-TPTZ (2,4,6-tripyridyl-s-triazine) complex to Fe^2^⁺-TPTZ; this complex produces an intense blue color with an absorption maximum at 593 nm. The reducing power is expressed as Fe^2^⁺ equivalents per gram of extract. This value is calculated using a calibration curve generated by preparing a series of known concentrations of FeSO_4_·7H_2_O (ferrous sulfate heptahydrate) solutions. The absorbance of these standard solutions is measured under the same assay conditions to establish a linear relationship between absorbance and Fe^2^⁺ concentration. The absorbance of the extract samples is then interpolated on the calibration curve to determine the Fe^2^⁺ equivalents. Finally, the results are normalized to the mass of the extract tested, providing the value of Fe^2^⁺ equivalents per gram of extract (mg Fe^2^⁺ equivalents/gram extract). Ascorbic acid was used as a standard. The analysis was performed in triplicate, with the results reported as mean ± SD.

### 3.5. Antibiofilm Activity

#### 3.5.1. Bacterial Strains and Minimal Inhibitory Concentration (MIC)

The bacterial strains used to assess antibacterial and antibiofilm activities included *Acinetobacter baumannii* (ATCC 19606), *Escherichia coli* (DSM 8579), *Pseudomonas aeruginosa* (DSM 50071), *Listeria monocytogenes* (ATCC 7644), and *Staphylococcus aureus* subsp. *aureus* Rosebach (ATCC 25923). The bacteria were cultured as previously reported [51]. The minimum inhibitory concentration (MIC) was calculated with the resazurin microtiter plate test in flat-bottomed 96-well plates, followed by 24 h incubation at 37 °C (35 °C for *A. baumannii*) [51]. Sterile DMSO served as the negative control, while tetracycline (7 µg/mL, dissolved in DMSO) was employed as the positive control. All determinations were performed in triplicate, and the data were reported as mean ± standard deviation.

#### 3.5.2. Crystal Violet Test

The effectiveness of the extracts in disrupting mature bacterial biofilms was evaluated using the method outlined by Fratianni et al., employing flat-bottomed 96-well microtiter plates (Falcon, VWR International, Milan, Italy), as previously described [51]. Biofilm adhesion was calculated as a percentage relative to the control, which consisted of bacterial cells grown without extracts (0% inhibition). Experiments were performed in triplicate, and data were reported as the mean ± SD.

#### 3.5.3. MTT Test

The efficacy of two concentrations (10 and 20 μL/mL) of the extracts on bacterial cell metabolic activity was determined employing the 3-(4,5-dimethylthiazol-2-yl)-2,5-diphenyltetrazolium bromide (MTT) colorimetric assay, as previously described [50]. The experiment was performed in triplicate, and the mean values were calculated to ensure reproducibility.

## 4. Conclusions

The chemical analysis of the hydroalcoholic extracts of three different *Mentha* species has been thoroughly characterized, revealing a diverse array of bioactive compounds, including flavonoids and phenolic acids, which may contribute to their therapeutic potential. Specifically, *M. longifolia* demonstrated moderate biofilm inhibition and antioxidant properties, while *M. spicata* exhibited selective antibacterial effects. Among these species, *M. pulegium* emerged as the most promising candidate, showcasing superior levels of phenolic and flavonoid compounds that significantly contributed to its potent antioxidant capability, as confirmed by DPPH and FRAP assays. Given these findings, the extracts can be considered for use in food preservation as natural additives to extend shelf life. Moreover, the pronounced antibacterial and biofilm-inhibiting properties of the *M. pulegium* extract, particularly against pathogens such as *Acinetobacter baumannii* and *Pseudomonas aeruginosa*, underscore its potential applications in the pharmaceutical industry.

## Figures and Tables

**Table 1:** LC-MS chemical composition of hydroalcoholic extracts of *M. longifolia*, *M. pulegium*, and *M spicata*. The numbers indicate the peak area of each compound.

No.	Family	Retention Time (Rt min)	Measured *m*/*z* [M + H]^+^	Molecular Formula	Δppm	Fragment	Fragment Formula	Fragment Ion (*m*/*z*)	Δppm	Identification	*M. longifolia*	*M.* *pulegium*	*M.* *spicata*
**1**	Phenolic acid	7.09	355.1021	C_16_H_18_O_9_	−0.700	[M-C_7_H_12_O_6_+H]^+^	C_9_H_7_O_3_	163.0387	−1.660	Chlorogenic acid	15,085.242	13,768.029	3206.164
						[M-C_7_H_12_O_6_-H_2_O+H]^+^	C_9_H_5_O_2_	145.0280	−2.661				
**2**	Oxomonocarboxylic acid	7.42	227.1275	C_12_H_18_O_4_	−1.037	[M-H_2_O+H]^+^	C_12_H_17_O_3_	209.1171	−0.578	Tuberonic acid	102,915.09	6120.2734	36,391.055
						[M-2H_2_O+H]^+^	C_12_H_15_O_2_	191.1066	−0.399				
						[M-C_2_(H_2_O)_2_+H]^+^	C_10_H_15_O_2_	167.1062	−2.910				
**3**	Flavone-*O*-glycoside	8.21	595.1647	C_27_H_30_O_15_	−1.708	[M-C_6_H_10_O_4_+H]^+^	C_21_H_21_O_11_	449.1080	0.294	Luteolin-7-*O*-Rutinoside	331,867.03	20,862.74	73,078.17
						[M-C_12_H_20_O_9_+H]^+^	C_15_H_11_O_6_	287.0552	0.576				
**4**	Flavone-*O*-glycoside	8.46	579.1694	C_27_H_30_O_14_	−2.421	[M-C_6_H_10_O_4_+H]^+^	C_21_H_21_O_10_	433.1116	−2.986	Apigenin 7-*O*-Rutinoside	4068.215	0	86,404.055
						[M-C_6_H_10_O_5_+H]^+^	C_21_H_21_O_9_	417.1164	−3.881				
						[M-C_12_H_20_O_9_+H]^+^	C_15_H_11_O_5_	271.0594	−2.730				
**5**	Flavone-*O*-glycoside	8.65	609.1811	C_28_H_32_O_15_	−0.405	[M-C_6_H_10_O_4_+H]^+^	C_22_H_23_O_11_	463.1234	−0.233	Diosmetin-7-*O*-rutinoside (Diosmina)	421,577.75	19,798.348	8247.895
						[M-C_12_H_20_O_9_+H]^+^	C_16_H_13_O_6_	301.0706	−0.148				
**6**	Flavone-*O*-glycoside	9.27	593.1859	C_28_H_32_O_14_	−0.964	[M-C_6_H_10_O_4_+H]^+^	C_22_H_23_O_10_	447.1282	−0.768	Acacetin 7-*O*-rutinoside (Linarin)	145,369.47	0	0
						[M-C_12_H_20_O_9_+H]^+^	C_16_H_13_O_5_	285.0754	−1.087				
**7**	Flavone-*O*-glycoside	9.38	595.2007	C_28_H_34_O_14_	−0.894	[M-C_12_H_20_O_9_+H]^+^	C_16_H_15_O_5_	287.0917	1.184	Isosakuranetin-*O*-rutinoside (Didymin)	n.d.	n.d.	n.d.
**8**	Caffeic acid dimer	9.56	341.0652	C_18_H_12_O_7_	−0.965	[M-H_2_O+H]^+^	C_18_H_11_O_6_	323.0551	0.419	Salvianolic acid G	9144.336	4414.9707	0
						[M-CO_2_+H]^+^	C_17_H_13_O_5_	297.0764	2.154				
						[M-C_9_H_6_O_4_+H]^+^	C_9_H_7_O_3_	163.0391	1.284				
						[M-C_9_H_6_O_5_+H]^+^	C_9_H_7_O_2_	147.0439	−0.993				
**9**	Flavonoid	9.87	287.0548	C_15_H_10_O_6_	−0.922	[M-H_2_O+H]^+^	C_15_H_9_O_5_	269.0428	−5.947	Kaempferol	159,260.27	16,393.633	48,949.184
						[M-(CH)_2_O+H]^+^	C_13_H_9_O_5_	245.0447	0.858				
						[M-C_8_H_6_O_2_+H]^+^	C_7_H_5_O_4_	153.0436	−1.275				
**10**	Flavonoid	10.35	331.0810	C_17_H_14_O_7_	−0.632	[M-CH_3_+H]^+^	C_16_H_12_O_7_	316.0580	0.809	Jaceosidin	164,605.33	0	0
						[M-CO+H]^+^	C_16_H_15_O_6_	303.0868	1.568				
						[M-C_2_H_3_O+H]^+^	C_15_H_12_O_6_	288.0623	−1.908				
**11**	Flavonoid	10.41	273.0757	C_15_H_12_O_5_	−0.037	[M-C_6_H_4_O_2_+H]^+^	C_9_H_9_O_3_	165.0544	−1.095	Naringenin	n.d.	n.d.	n.d.
						[M-C_7_H_11_O_3_+H]^+^	C_6_H_2_O_2_	130.0039	−3.083				
**12**	Flavonoid	10.48	361.091	C_18_H_16_O_8_	−0.509	[M-CH_3_+H]^+^	C_17_H_14_O_8_	346.0681	−0.689	Sideritiflavone	335,510.84	4927.666	523,152.12
						[M-_2_CH_3_+H]^+^	C_16_H_11_O_8_	331.0445	−1.294				
						[M-CH_5_O+H]^+^	C_17_H_12_O_7_	328.0576	−0.409				
**13**	Flavonoid	10.59	301.0704	C_16_H_12_O_6_	−0.779	[M-CH_3_+H]^+^	C_15_H_10_O_6_	286.0471	−0.348	Diosmetin	797,023.06	8806.57	222,210.06
						[M-CH_3_-CO+H]^+^	C_14_H_10_O_5_	258.0523	0.252				
**14**	Flavonoid	10.87	345.0960	C_18_H_16_O_7_	−2.519	[M-CH_3_+H]^+^	C_17_H_14_O_7_	330.0727	−2.073	Xanthomicrol	89,237.6	83,810.945	33,379.188
						[M-C_2_H_4_O+H]^+^	C_16_H_13_O_6_	301.0701	−1.975				
**15**	Flavonoid	10.90	361.0912	C_18_H_16_O_8_	−1.672	[M-CH_3_+H]^+^	C_17_H_14_O_8_	346.0682	−0.401	Thymonin	640,358.6	3886.9512	99,089.83
						[M-_2_CH_3_+H]^+^	C_16_H_11_O_8_	331.0445	−0.948				
						[M-CH_5_O+H]^+^	C_17_H_12_O_7_	328.0576	−0.409				
						[M-C_2_H_8_O+H]^+^	C_16_H_9_O_7_	313.0340	−0.796				
**16**	Flavonoid	11.33	375.1069	C_19_H_18_O_8_	−1.370	[M-CH_3_+H]^+^	C_18_H_16_O_8_	360.0841	0.392	5,7-Dihydroxy-6,8,3′,4′-tetramethoxyflavone (Hymenoxin)	513,052.94	12,278.242	100,773.44
						[M-C_2_H_6_+H]^+^	C_17_H_13_O_8_	345.0608	0.945				
						[M-CH_5_O+H]^+^	C_18_H_14_O_7_	342.0735	0.163				
						[M-C_3_H_5_O_4_+H]^+^	C_16_H_14_O_4_	270.0887	0.332				
						[M-C_10_H_10_O_5_+H]^+^	C_9_H_9_O_3_	165.0548	1.329				
**17**	Flavonoid	11.75	403.1376	C_21_H_22_O_8_	−2.838	[M-CH_3_+H]^+^	C_20_H_20_O_8_	388.1154	0.466	Nobiletin	1682.9082	15,424.356	6241.834
						[M-C_2_H_6_+H]^+^	C_19_H_17_O_8_	373.0897	−0.720				
**18**	Flavonoid	11.83	287.0916	C_16_H_14_O_5_	−0.836	[M-C(H_2_O)_3_+H]^+^	C_10_H_9_O_2_	161.0596	−0.100	Sakuranetin	16,995.594	0	1518.3528
						[M-(C_8_H_8_O)+H]^+^	C_8_H_7_O_4_	167.0342	2.064				
**19**	Flavonoid	11.89	345.0963	C_18_H_16_O_7_	−1.737	[M-CH_3_+H]^+^	C_17_H_14_O_7_	330.0738	1.260	Nevadensin	528,516.4	306,378.25	23,202.852
						[M-(CH_3_)_2_+H]^+^	C_16_H_11_O_7_	315.0504	1.431				
						[M-(CH_5_O)+H]^+^	C_17_H_12_O_6_	312.0633	1.540				
						[M-C_10_H_10_O_5_+H]^+^	C_8_ H_7_O_2_	135.0440	−0.489				
**20**	Flavonoid	12.33	359.1122	C_19_H_18_O_7_	−0.889	[M-CH_3_-H_2_O+H]^+^	C_18_H_14_O_6_	326.0788	0.860	5-Hydroxy-6,7,3′,4′-tetramethoxy flavone	0	131,156.69	0
						[M-CH_3_+H]^+^	C_18_H_16_O_7_	344.0893	0.865				
						[M-H_2_O+H]^+^	C_19_H_17_O_6_	341.1017	−0.923				
						[M-C_2_H_4_O+H]^+^	C_17_H_15_O_6_	315.0871	2.683				
						[M-C_2_H_5_O_2_+H]^+^	C_17_H_14_O_5_	298.0840	1.359				
**21**	Flavonoid	12.78	389.1227	C_20_H_20_O_8_	−2.477	[M-CH_3_+H]^+^	C_19_H_18_O_8_	374.0990	−0.589	5-Hydroxyauranetin	74,619.89	17,551.996	18,330.71
						[M-C_2_H_6_+H]^+^	C_18_H_15_O_8_	359.0758	−0.846				
						[M-CH_3_-H_2_O+H]^+^	C_19_H_16_O_7_	356.0883	−0.764				
						[M-C_2_H_6_-H_2_O+H]^+^	C_18_H_13_O_7_	341.0650	−1.786				
						[M-C_2_H_3_O_2_+H]^+^	C_18_H_16_O_6_	328.0930	−1.120				

n.d.: not determined on the basis of the set parameters. See the text for explanation.

**Table 2:** Antioxidant activity of *M. pulegium*, *M spicata*, and *M. longifolia*.

	*M. longifolia*	*M. pulegium*	*M. spicata*	Ascorbic Acid
**DPPH** **^1^ IC_50_(µg/mL)** **^2^ Mean (±SD)**	16.42 (±0.10) ^b^	16.13 (±2.03) ^b^	29.26 (±0.35) ^c^	2.98 (±0.35) ^a^
**FRAP** **Emg Fe^2+^ Equivalents/g Extract** **^2^ Mean (±SD)**	181.89 (±6.88) ^ab^	272.36 (±46.02) ^b^	104.45 (±21.99) ^a^	577.77 (±63.50) ^c^

^1^ IC_50_ = the concentration needed to decrease the absorbance of DPPH by 50%. ^2^ Mean (±SD) = represents the average value of the three tests and the standard deviation. Ascorbic acid is employed as the reference standard in antioxidant analyses. Means followed by different letters in the same row indicate significant differences at *p* < 0.05 (two-way ANOVA followed by Tukey’s post hoc test).

**Table 3:** MIC of *M. longifolia*, *M. pulegium*, and *M. spicata* extracts (µL/mL). The results are presented as the mean (±SD) of three independent experiments. Tetracycline (7 µg/mL) was used as the control. ^a^: *p* < 0.05; ^b^: *p* < 0.01 (ANOVA followed by Dunnett’s multiple comparison test).

MIC	*M. longifolia*	*M. pulegium*	*M. spicata*	Tetracycline
*A. baumannii*	38.00 (±1.00) ^a^	38.00 (±2.00) ^a^	36.00 (±2.00) ^a^	30.00 (±1.00)
*E. coli*	>50.00 ^b^	>50.00 ^b^	>50.00 ^b^	30.00 (±2.00)
*L. monocytogenes*	>50.00 ^b^	42.00 (±1.00) ^a^	>50.00 ^b^	28.00 (±1.00)
*P. aeruginosa*	42.00 (±1.00) ^a^	46.00 (±2.00) ^a^	42.00 (±1.00) ^a^	28.00 (±2.00)
*S. aureus*	>50.00 ^b^	>50.00 ^b^	36.0 (±2.00) ^a^	34.00 (±2.00)

Means followed by different letters in the same row indicate significant differences at *p* < 0.05 (two-way ANOVA followed by Tukey’s post hoc test).

**Table 4:** Biofilm inhibitory activity (CV) and inhibitory activity against the sessile cells’ metabolism (MTT test), expressed as percentage, of the extracts of the three *Mentha* species, added at 10 and 20 μL/mL after 24 h of incubation against the pathogens *Acinetobacter baumannii*, *Escherichia coli*, *Listeria monocytogenes*, *Pseudomonas aeruginosa*, and *Staphylococcus aureus*. The results are presented as the mean of three independent experiments (±SD). ^a^: *p* < 0.05; ^b^: *p* < 0.01 (ANOVA followed by Dunnett’s multiple comparison test).

CV	*M. longifolia* 10	*M. longifolia* 20	*M. pulegium* 10	*M. pulegium* 20	*M. spicata* 10	*M. spicata* 20
*A. baumannii*	44.38 (±0.97) ^b^	47.61 (±0.89) ^b^	42.95 (±1.09) ^b^	45.63 (±2.08) ^b^	46.38 (±0.72) ^b^	50.78 (±1.92) ^b^
*E. coli*	0.00 (±0.00)	0.00 (±0.00)	0.00 (±0.00)	0.00 (±0.00)	0.00 (±0.00)	0.00 (±0.00)
*L. monocytogenes*	0.00 (±0.00)	0.00 (±0.00)	0.00 (±0.00)	21.97 (±0.51) ^a^	0.00 (±0.00)	0.00 (±0.00)
*P. aeruginosa*	0.00 (±0.00)	22.67 (±1.80) ^a^	0.00 (±0.00)	5.22 (±0.72) ^a^	0.00 (±0.00)	21.54 (±0.18) ^a^
*S. aureus*	0.00 (±0.00)	0.00 (±0.00)	0.00 (±0.00)	0.00 (±0.00)	0.00 (±0.00)	0.00 (±0.00)
**MTT**	***M. longifolia* 10**	***M. longifolia*** **20**	***M. pulegium*** **10**	***M. pulegium* 20**	***M. spicata* 10**	***M. spicata* 20**
*A. baumannii*	0.00 (±0.00)	0.00 (±0.00)	0.00 (±0.00)	17.30 (±1.18) ^a^	0.00 (±0.00)	0.00 (±0.00)
*E. coli*	0.00 (±0.00)	0.00 (±0.00)	0.00 (±0.00)	0.00 (±0.00)	0.00 (±0.00)	0.00 (±0.00)
*L. monocytogenes*	0.00 (±0.00)	0.00 (±0.00)	0.00 (±0.00)	0.00 (±0.00)	6.02 (±0.21) ^a^	37.71 (±3.31) ^b^
*P. aeruginosa*	0.00 (±0.00)	0.00 (±0.00)	0.00 (±0.00)	15.25 (±1.34) ^a^	0.00 (±0.00)	28.34 (±2.09) ^a^
*S. aureus*	0.00 (±0.00)	0.00 (±0.00)	0.00 (±0.00)	9.51 (±0.67) ^a^	0.00 (±0.00)	0.00 (±0.00)

Means followed by different letters in the same row indicate significant differences at *p* < 0.05 (two-way ANOVA followed by Tukey’s post hoc test).

## Data Availability

The original contributions presented in this study are included in the article/Supplementary Materials. Further inquiries can be directed to the corresponding author(s).

## Supplementary Materials

The following supporting information can be downloaded at: https://www.mdpi.com/article/10.3390/plants14030360/s1. Table S1: Parameters chosen for MZmine software.
