# Supplementary material for: Chemical Profile and Bioactivities of Three Species of Mentha Growing in the Campania Region, Southern Italy
Source: Plants (Basel). 2025 Jan 24;14(3):360. doi: 10.3390/plants14030360 (PMC11821235; doi:10.3390/plants14030360)
Supplement: Supplementary file 1 [file plants-14-00360-s001.zip › plants-3383376-supplementary.pdf]

Table S1. Parameters chosen for MZmine software

**Mass Detection**	
Noise level MS1,level=1	3.0E2
Noise level MS2,level=2	3.0E2
Mass detector	centroid
**ADAP Chromatogram Builder**	
Min consecutive scans	5
Min intensity for consecutive scans	1.00E+03
Min absolute height	1.0E+03
scan to scan accuracy (m/z)	0.003
**Smoothing**	
Savitzky Golay	
retention time smoothing	5
**Local minimum feature resolver**	
Dimension	Retention Time
Chromatographic threshold	85%
Minimum search range RT/Mobility (absolute)	0.05
Minimum relative height:	0%
Minimum absolute height	1.00E+03
min ratio of peak top/edge	2.0
peak duration range	0.0-2.0
min # of data points	5
**13C isotope filter**	

m/z tolerance	0.0015
retention time tolerance	0.05
monotoninc shape	yes
maximum charge	1
representative isotope	most intense
never remove feature with MS2	yes
**Isotopic peaks finder**	
chemical elements	H,C,N,O,S
m/z tolerance	0.0030
maximum charge	1
search in scans	single most intense
**Join aligner**	
m/z tolerance	0.005
retention time tolerance	0.3
weight for m/z	1
mobility weight	1
**Peak finder**	
Intensity tolerance	80%
m/z tolerance (m/z)	0.005
retention time tolerance	0.3
minimum data points	5
